# A soft stretchable bending sensor and data glove applications

**DOI:** 10.1186/s40638-016-0051-1

**Published:** 2016-12-01

**Authors:** Zhong Shen, Juan Yi, Xiaodong Li, Mark Hin Pei Lo, Michael Z. Q. Chen, Yong Hu, Zheng Wang

**Affiliations:** 1 Department of Mechanical Engineering, The University of Hong Kong, Pok Fu Lam, Hong Kong SAR China; 2 HKU Shenzhen Institute of Research and Innovation, Shenzhen, China; 3 Department of Orthopaedics and Traumatology, The University of Hong Kong, Pok Fu Lam, Hong Kong SAR China

## Abstract

Soft sensors are required to accommodate the flexible and deformable natures of the human body in wearable device applications. They are also suitable for integration with soft robotic devices to monitor the performance status and provide references for feedback control. However, the choices for bending sensors are still highly limited. In this paper, a soft bending sensor is presented. By careful design with a blend of sensitive and insensitive regions, the sensor could be stretchable while being insensitive to stretching. An analytical study was presented on how to design the sensor with the named bending/stretching feature. This feature enables the sensor to be implemented in measuring human motions where a large amount of skin stretch is involved. Two sensor gloves were designed and fabricated based on the proposed soft bending sensor, aiming for different application scenarios. Both the sensor and the gloves were evaluated using a dedicated evaluation platform with experimental results compared against each other.

## Introduction

Soft sensors are receiving growing attention, due to both the global wave of developments in wearable human-centered devices and the recent focus on soft robots [1–4]. For human motions, soft sensors could provide direct joint-level angle measurements, hence lead to a reconstruction of human body trajectories. For soft robots, soft sensors are required to provide sensory and control information while not interfering with the primary compliant and adaptive features of the robotic devices. However, for both of the above application scenarios, bending and stretch are two closely coupled factors. It is technically very challenging to make stretchable bending sensors insensitive to stretching. The available flexible bending sensors are very limited, with few allowing for stretching.

In the last two decades, many researchers have engaged in developing a new kind of sensing technology, whether in hardware or software, to meet the need of soft wearable devices. Optical fiber sensors, which have been recognized as a standard for motion capture, have drawn some attention [5–7]. Apart from optical fiber sensors, there were other groups making similar wearable motion capture devices using resistive bending sensors [8–10] and using a filter and multiple sensors for one joint to reduce the error [11]. Except resistive sensors, printed sensors or MEMS sensors can also provide small size, low cost and flexibility [12, 13]. Recently, a new kind of sensors that uses a conductive liquid metal injected in a soft chamber has drawn much attention. The resistance of the sensor will change as the shape of the chamber deforms due to external force [14–18]. This soft stretchable bending sensor suits well for wearable devices, but the fluidity of liquid metal still brings technical constraints and limits its practiced application.

**Fig. 1:**
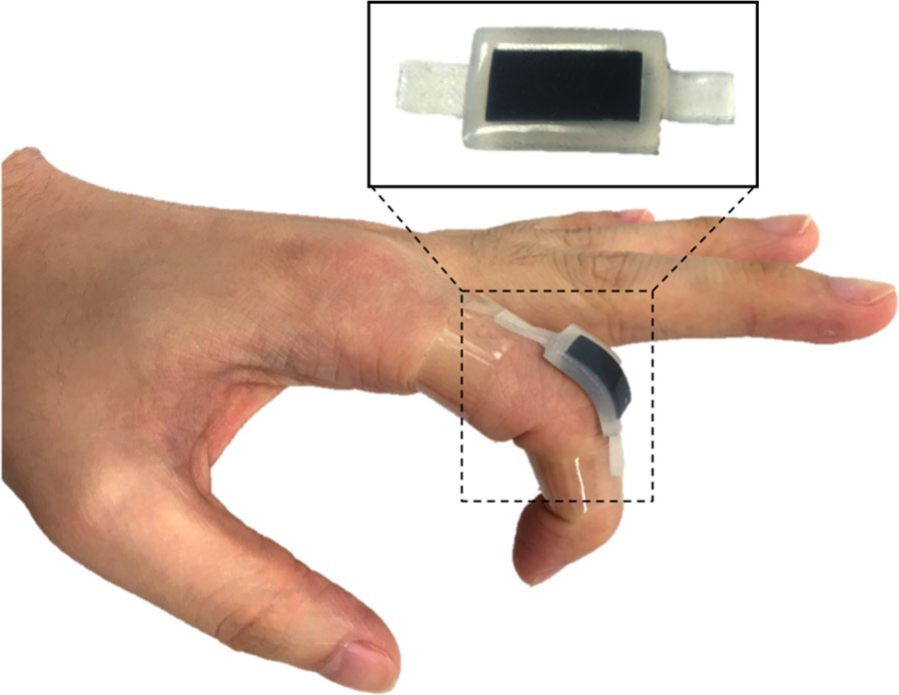
Illustration of a soft stretchable bending sensor mounted on an adult’s hand. The sensor uses EPR embedded in a soft stretchable base. The soft and stretch abilities of the sensor allow it to attach firmly on the finger joint

The techniques mentioned above have contributed to the wearable sensing technologies. For wider and further applications, bending or strain sensors are still in need of flexibility, comfort and accuracy as well as low cost and non-toxicity. So, here we present a novel low-cost soft stretchable bending sensor (Fig. 1) along with two different sensor gloves. Both the sensor and the gloves have excellent flexibility as well as precision. The sizes of the sensor and the gloves are also customizable, which is a key issue for wearable devices.

In “Sensor and sensor glove” section, the design details of the bending sensor and two kinds of sensor gloves are described. “Sensor evaluation” section illustrates the experimental methods and results for the bending sensor. “Glove evaluation” section shows the testing results for two kinds of sensor gloves. “Conclusions” section presents the conclusion and future work.

## Sensor and sensor glove

### Sensor design

Bending fingers means stretched surface, but most existing sensors are non-stretchable. The sensor’s slipping on skin may cause serious problems and increase error. In this case, we design a stretchable bending sensor that is insensitive to stretching.

**Table 1:** Typical properties of the sensing material

Properties	Temperature rating	Tensile strength	Elongation	Volume resistivity	Weight	Power consumption
Typical value	$194^{\circ }$	6 lbs/in	800%	$10^3$ Ω-cm	0.16 g cm$^{-3}$	<0.1 W

**Fig. 2:**
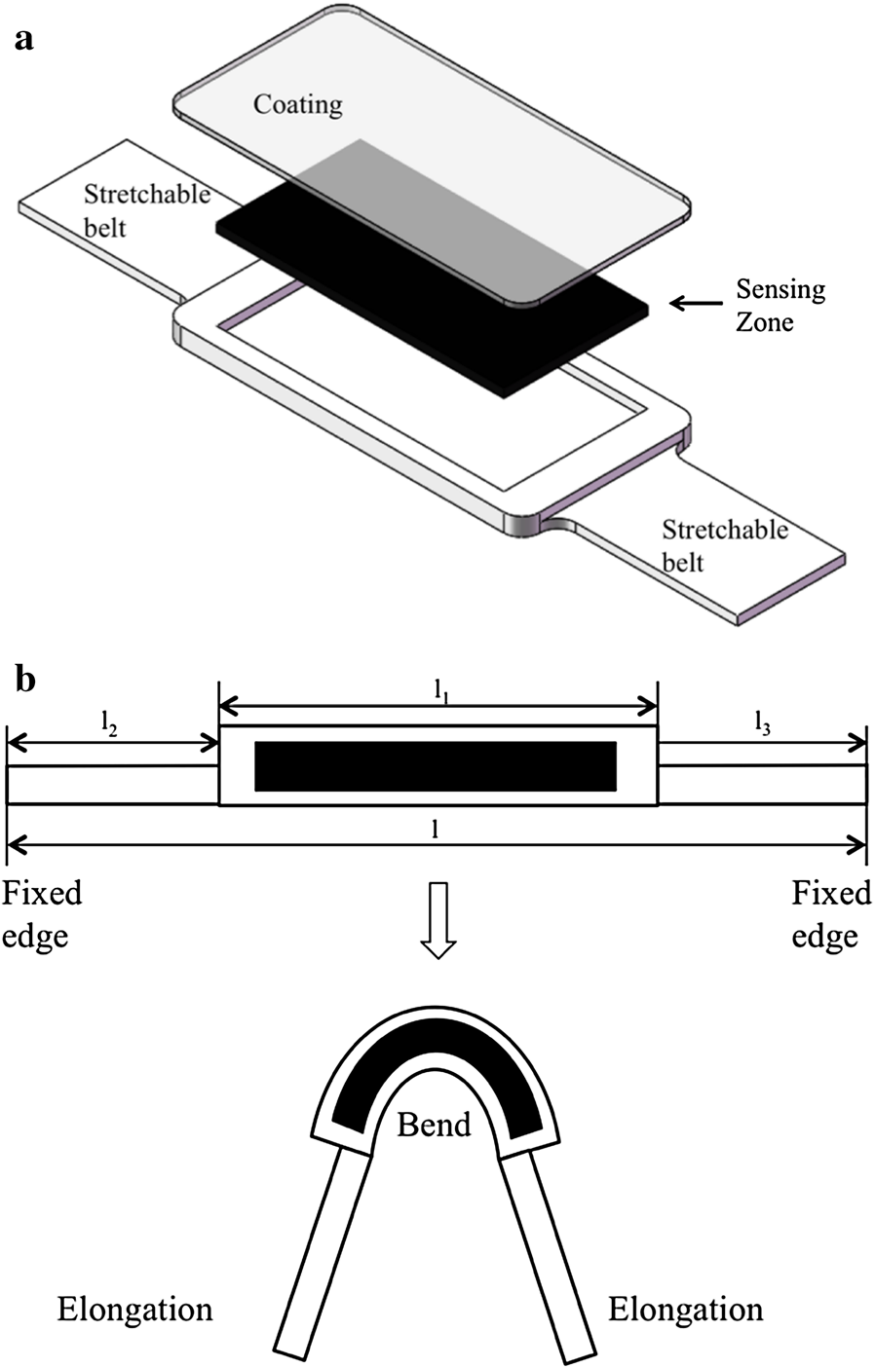
**a** Sensor structure. The middle part is designed to be thicker and wider than the two belts, offering better stiffness to avoid stretching. **b** Profile chart of the sensor. The middle part, which contains the sensing material, will bend with permitted stretch. Elongation caused by finger bend will be offset by the stretch of the two belts

The sensors are based on ethylene propylene rubber (EPR). We used the Scotch Electrical Semi-Conducting Tape 13 (3M Company, Maplewood, Minnesota, USA) as the sensing material. When the material is bent or stretched, the resistance will change. Typical physical and electrical properties of the material are shown in Table 1. EPR will have elastic deformation when elongation is less than 2% [19], but the elongation of stretched skin may reach 40%, which may cause serious plastic deformation and change the resistance permanently. In order to make the sensor stretchable, a structure is needed to separate stretch from bending. We settled on a soft housing for the EPR portion. The whole structure is consisted of three parts: two belts and the middle part (Fig. 2). Considering the length and elongation of the sensor:

1 $$l= l_1+l_2+l_3$$

2 $$\Delta l= \Delta l_1+\Delta l_2+\Delta l_3$$

3 $${\mathcal {E}}_hl= {\mathcal {E}}_1l_1+{\mathcal {E}}_2l_2+{\mathcal {E}}_3l_3$$

*l* is the length of the sensor in (1) and (3), $\Delta l$ is the elongation and ${\mathcal {E}}_h$ is the maximum strain caused by finger bend. Using Hooke’s law, we have:

4 $$f=a_iE_il_i{\mathcal {E}}_i \quad (i=1, 2, 3)$$

*f* is the force applied on the sensor, *a* is the cross-sectional area and E is the Young’s modulus. Thus,

5 $${\mathcal {E}}_1l_1=\frac{a_2E_2l_2}{a_1E_1}{\mathcal {E}}_2,\quad {\mathcal {E}}_3l_3=\frac{a_2E_2l_2}{a_3E_3}{\mathcal {E}}_2$$

Substituting (5) into (3), we have:

6 $${\mathcal {E}}_hl=\sum _{i=1,2,3}\left( \frac{1}{a_iE_i}\right) a_2E_2l_2{\mathcal {E}}_2$$

Thus,

7 $${\mathcal {E}}_2=\frac{{\mathcal {E}}_hl}{a_2E_2l_2}\left( \sum _{i=1,2,3} \left( \frac{1}{a_iE_i}\right) \right) ^{-1}$$

Eq. (7) provides a method of determining the geometric design of the sensor to ensure the elongation of the sensor section is always within the repeatable region.

**Fig. 3:**
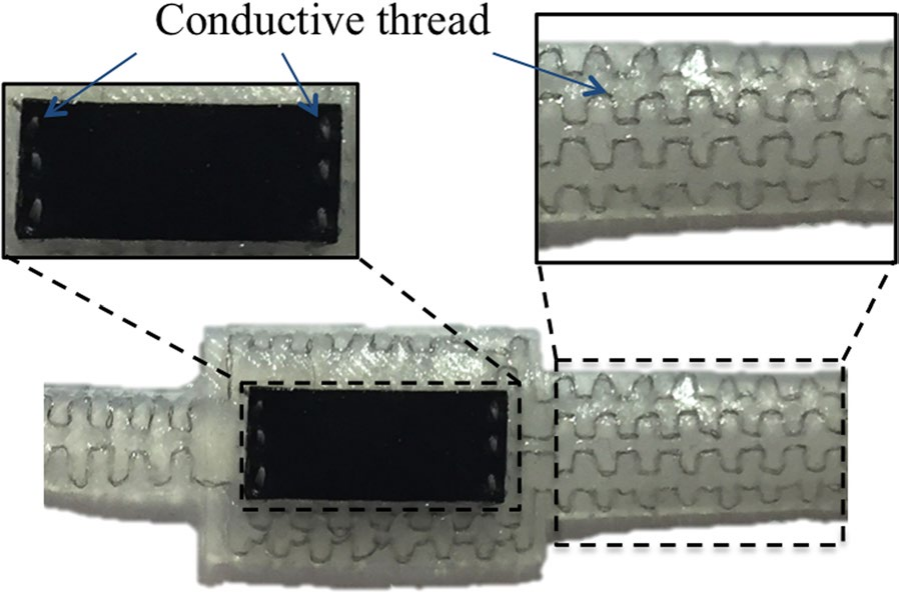
Connection method for EPR sensor. A sliver-plated nylon thread is used to connect the sensor to the circuit. A certain length has been reserved in the soft belts to prevent the thread from breaking when the belts are stretched

A soft rubber called Ecoflex 00-30(Smooth-On, East Texas, PA 18046) was used to mold the sensor, and three steps are needed to fabricate a sensor. First the main structure at the bottom is molded. Then, the sensing material is put in the middle of the structure and connected to the circuit. In order to maintain the soft ability of the sensor, a sliver-plated nylon thread(Less EMF Inc, Latham, NY, USA, Cat.#A1226) is used to connect the sensor to the circuit. As shown in Fig. 3, the thread is first sewed into the two distal end of the sensor and then sewed into the soft belts. Because the nylon thread is not stretchable, we reserved some length in the soft belts to prevent conductive thread from breaking when the belts were stretched. After the connection process, a coating layer is added on the sensing material to seal up the sensing material.

### Fabric data-collecting glove

**Fig. 4:**
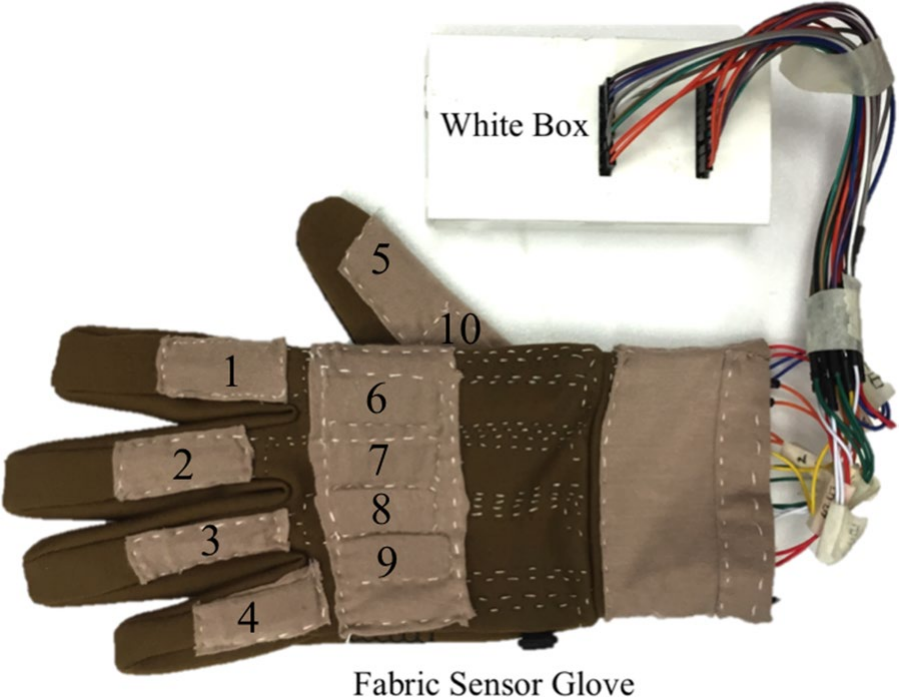
Fabric data-collecting glove. In total 10 soft stretchable sensors were integrated in the glove. The *white box*, which contains the electrical components, is used to connect the glove to the computer

Hand posture is one of the most representative and complicated motion for human body motion capture. Here, we present a sensor glove for monitoring hand posture as well as testing the sensor (Fig. 4).

Most of the existing bending sensors are non-stretchable, and they will slip when fingers bend. So traditional data gloves have to be made larger than the hand size to reserve some space for the sensors to slip. The gap between the glove and hand may cause some significant error. For the fabric data-collecting glove, the glove size can be fully customized due to the stretch ability of the EPR-based bending sensor. Each sensor can be fixed on the fabric glove without slipping so the error caused by sensor slipping can be minimized.

In sum, there are 10 bending sensors used in the sensor glove and all the resistance are measured using Arduino Mega 2560 in the white box. Two sensors are used on the thumb to measure metacarpophalangeal joint (MP) and distal interphalangeal joint (DIP). Except for the thumb, two sensors are used for each finger to measure the bending angle of MP joint and proximal interphalangeal (PIP) joint; DIP joints are not measured. Since there is a linkage between the proximal and distal interphalangeal joints, DIP joints cannot bend independently [20].

### Soft rubber data-collecting glove

**Fig. 5:**
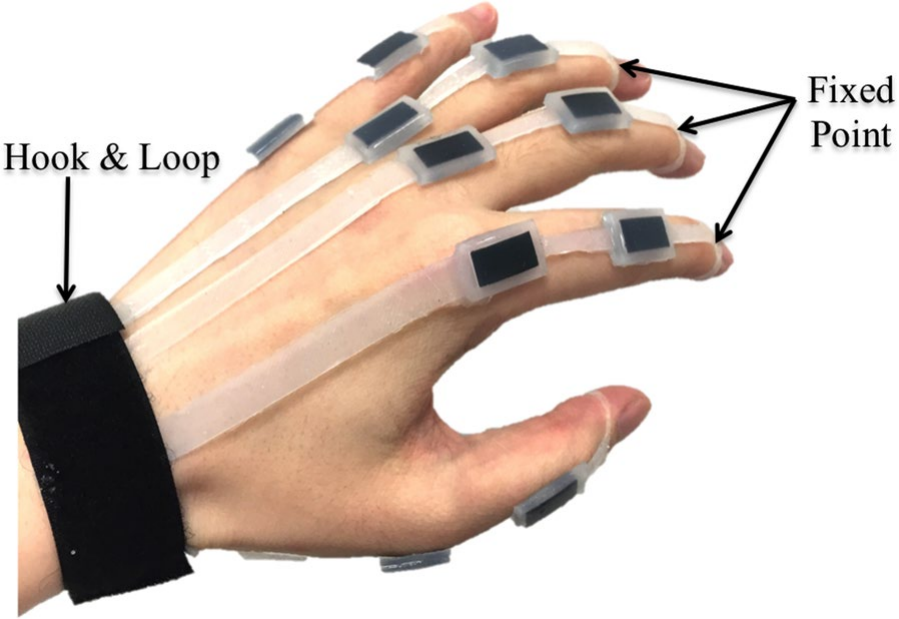
Soft rubber data-collecting glove. The top of each part of the glove is a ring-shaped structure, and it is used to locate one end of the glove on the fingertips. The bottom of the five parts fixed inside the hook&loop to locate the other end of the glove on the wrist. There is a preload in each part of the glove to keep the sensing zones on the corresponding joints

Most existing data-collecting gloves are designed and fabricated based on traditional fabric gloves. The size of the glove is fixed, and normally the glove does not fit well for different individuals and the complex structure of data gloves will affect the precision. In order to avoid these problems, a new modular data-collecting glove is developed for hand posture capture (Fig. 5).

The novel data-collecting glove uses soft rubber as the base material instead of traditional fabric gloves. The soft stretchable structure makes it possible for providing the same action like the skin. The glove is made of five parts representing five fingers, and all parts have the same structure. For each part, there are two sensing zones and three connecting belts. The belt in the middle connects the two sensing zones, and the length of the belt is various to fit in with different finger sizes. The other belts are used to locate the glove on a hand. Due to the stretch ability of the soft rubber, the glove can be easily adjusted to adapt to different hand sizes and each sensor can be precisely mounted on the corresponding finger joint. In fact, this method can be used for multiple joints measurement. Assuming that for each part there are *n* sensing zones and $n+1$ connecting belts, the total elongation should be:

8 $${\mathcal {E}}L=\sum _{i=1}^{n}({\mathcal {E}}_{si}L_{si} + {\mathcal {E}}_{ci}L_{ci})+{\mathcal {E}}_{c(n+1)}L_{c(n+1)}$$

*L* is the length of each part and ${\mathcal {E}}$ is the strain of each part. Using Hooke’s law, we have:

9 $$\begin{aligned}{\mathcal {E}}_{ci}L_{ci}&=\frac{A_{si}E_{si}L_{si}{\mathcal {E}}_{si}}{A_{ci}E_{ci}} \quad (i=1,2,3,\ldots ,n),\nonumber \\{\mathcal {E}}_{c(n+1)}L_{c(n+1)}&=\frac{A_{sn}E_{sn}L_{sn}{\mathcal {E}}_{sn}}{A_{c(n+1)}E_{c(n+1)}} \end{aligned}$$

*A* is the cross-sectional area and *E* is the Young’s modulus. Substituting (9) into (8),

10 $$\begin{aligned} {\mathcal {E}}_{sn}&= \left( {\mathcal {E}}L-\sum _{i=1}^{n-1} \left( \frac{A_{si}E_{si}L_{si}{\mathcal {E}}_{si}}{A_{ci}E_{ci}} +{\mathcal {E}}_{si}L_{si}\right) \right) \nonumber \\&\quad\left( L_{sn}+\frac{A_{sn}E_{sn}L_{sn}}{A_{cn}E_{cn}} +\frac{A_{sn}E_{sn}L_{sn}}{A_{c(n+1)}E_{c(n+1)}}\right) ^{-1} \end{aligned}$$

Eq. (10) provides a method of determining the geometric design of each sensing zone to ensure the elongation of the sensor section is always within the repeatable region. Compared with traditional data gloves, this glove acts just like the skin on hand so it can directly measure the bending motion without any intermediary. And the soft modular design of the glove greatly expands the scope of applications such as nuclear magnetic resonance imaging(MRI) compatible scenarios.

## Sensor evaluation

### Sensitivity

**Fig. 6:**
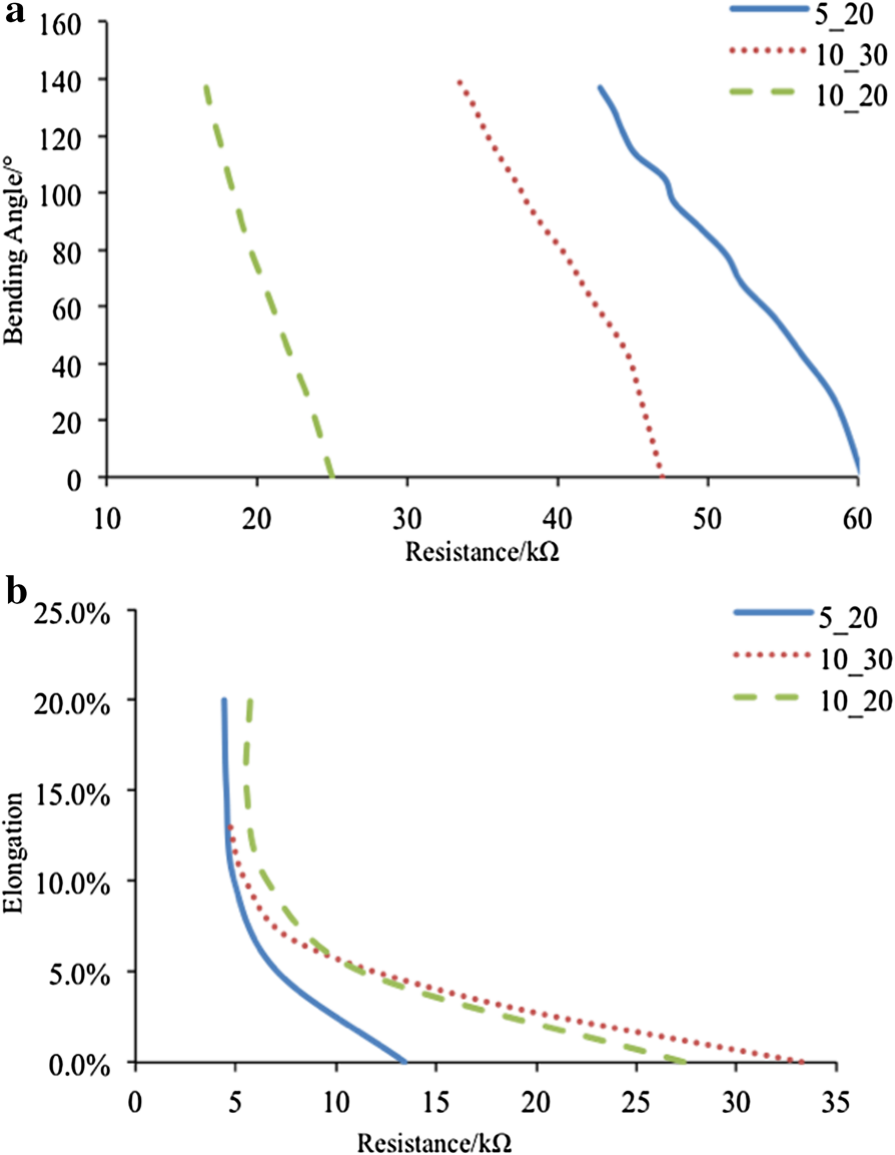
Testing results of the sensor’s sensitivity to bend and stretch. **a** Sensitivity to bend. **b** Sensitivity to stretch. Unrecoverable plastic deformation will take place when elongation is up to 2%. Three different sizes are designed for both tests, and their width-to-length ratio is all set to 1:2, 1:3 and 1:4 for comparison

Bending fingers always comes up with two portions: the bending motion and the skin stretch. Either portion can be regarded as a measurement object. Thus, the sensing material’s sensitivity to bend and stretch is tested separately (Fig. 6). Three different sizes with respect to length-to-width ratio are designed to explore the relationship between sensor size and sensitivity.

The results illustrate that the sensor exhibits excellent sensitivity to both bend and stretch. For bending motion, the resistance changing ranges for the size of $5\times 20\,\hbox {mm}, 10\times 30\,\hbox {mm}$ and $10\times 20\,\hbox {mm}$ are 30.6, 29.2 and 33.52%, respectively. All the three designs show similar sensitivity to bend, and the sensitivity to bend is independent to sensor size. For stretching, the sensor of size $10\times 30\,\hbox {mm}$ and $10\times 20\,\hbox {mm}$ exhibit similar sensitivity (86.0 and 79.8%) and the sensor of size $5\times 20\,\hbox {mm}$ has a resistance changing range of 67.1%, illustrating the sensitivity to stretch is related to the width of the sensor.

### Repeatability

**Fig. 7:**
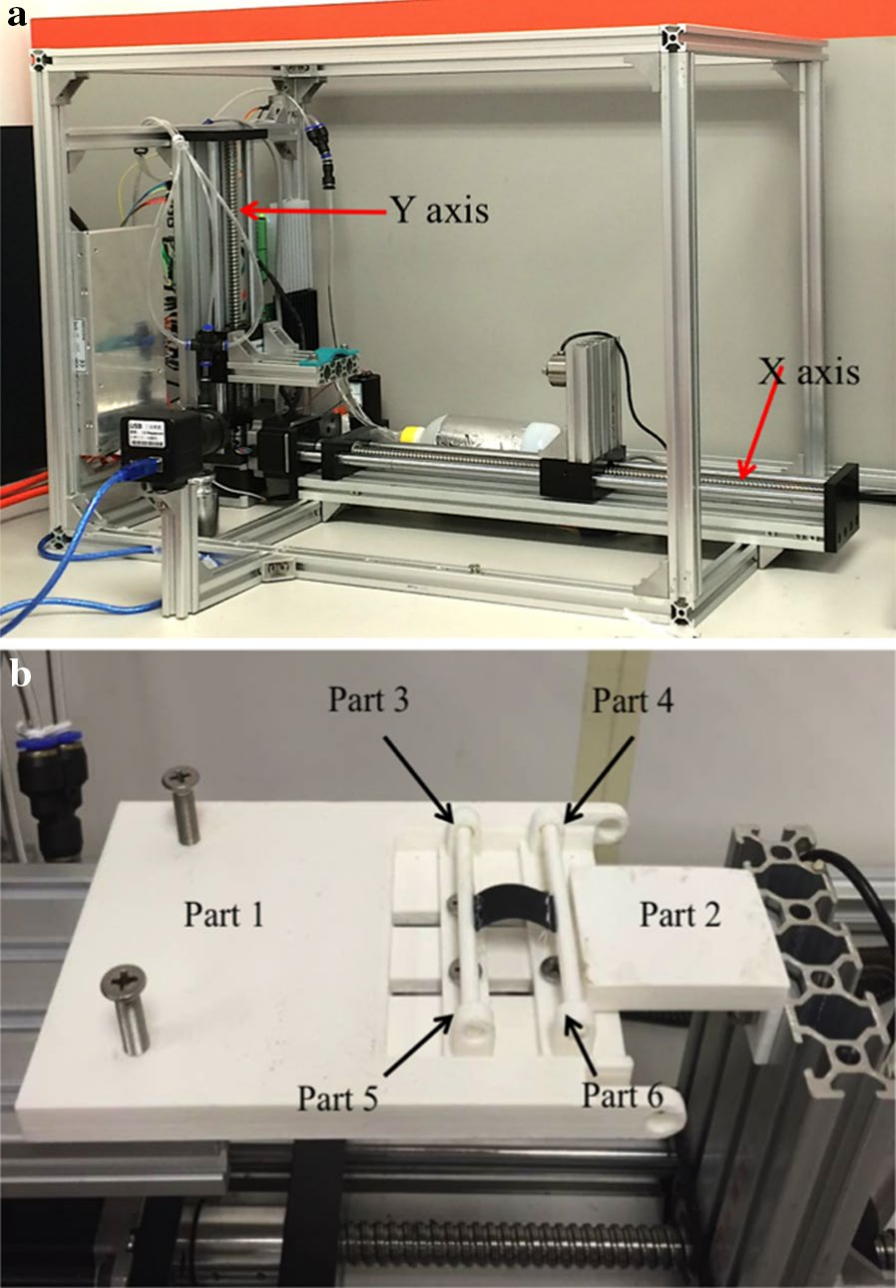
**a** Testing platform. Both axes are driven by stepper motor, and the minimum displacement is 1 mm. **b** Testing structure used to transform linear movement into bending motion. The black part is the sensor separated from the stretchable structure

The sensing material is sensitive to both bend and stretch. But stretching will cause unrecoverable plastic deformation, and the resistance will change permanently as well. By using the soft structure mentioned in “Sensor and sensor glove” section, the sensing material’s stretching is avoided and repeatability of bending is tested. In order to test the sensor’s repeatability apart from the soft structure, a dedicated evaluation platform was developed. The platform has two-degrees-of-freedom motion in the horizontal and vertical movements. To test the repeatability of the sensor, a highly repeatable and precise testing procedure is in need. The procedure should also be simple to minimize the inherent error. In this case, we produce a specific structure to test the sensor (Fig. 7).

**Table 2:** Testing results of fabric data-collecting glove

Finger	Index finger	Middle finger	Ring finger	Little finger	Thumb
SD	4.20	3.73	6.46	4.93	6.32

**Fig. 8:**
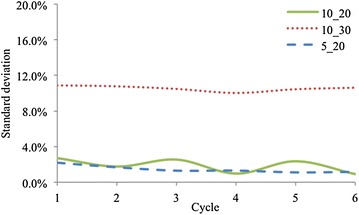
Testing results of repeatability for three different sizes of sensors, illustrating good repeatability for the size of $10\times 20$ mm and $5\times 20$ mm. The testing results for the size of $10\times 30$ mm illustrate that the length of the sensor should not exceed 20 mm

**Fig. 9:**
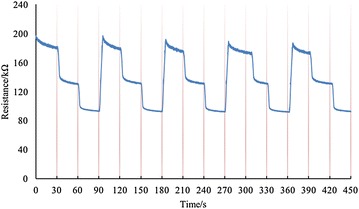
Drifting test results. The sensor is kept still in each angle for 30 s. The resistance drops about 8.5% when the sensor is flat and drops 3.1 and 1.5% when then bending angle is 60$^{\circ }$ and 120$^{\circ }$ respectively

**Fig. 10:**
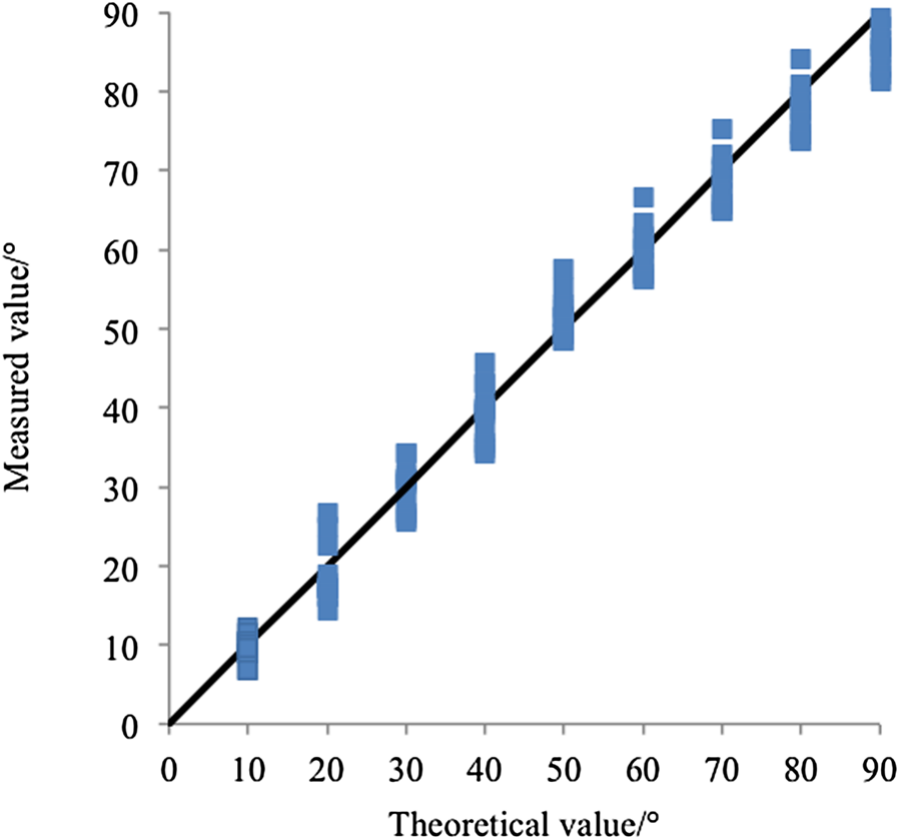
Testing results of Flex Sensor 2.2. In total nine angles are tested, and for each angle, ten values are measured. The measured values closely distribute around the *black line*, illustrating good accuracy of the sensor

The structure consists of 6 parts. Sensor is the black part. There are notches on Part 3 and Part 4, which are used to fix the sensor’s two edges. The rotation of these two parts can avoid bending on two edges. The sensor is flat in the beginning. Assume the length of the sensor is *l*. When Part 6 moves $\Delta l$, the sensor will be bent in the middle. Consider the bent sensor as an arc, the radian should be the bending angle of the sensor. As the length of the sensor is a constant, we have:

11 $$l = \theta R$$

*R* is the radius of the arc. From the isosceles triangle, we have:

12 $$\cos \theta = 1 - \frac{(l-\Delta l)^2}{2R^2}$$

Then we have:

13 $$\cos \theta = 1 - \frac{(l-\Delta l)^2}{2\frac{l^2}{\theta ^2}}$$

**Fig. 11:**
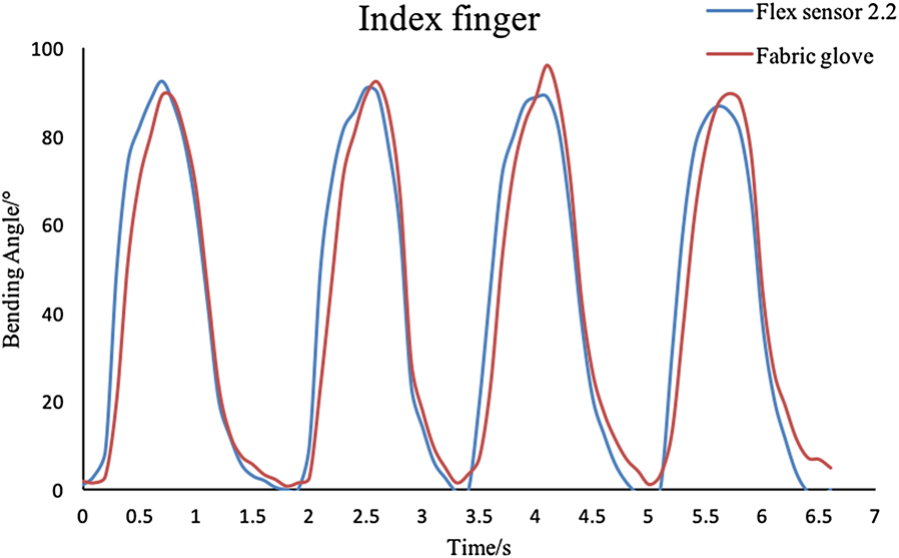
Testing results of PIP joint of the fabric data-collecting glove. The two lines match quite well, illustrating excellent accuracy as well as repeatability

**Fig. 12:**
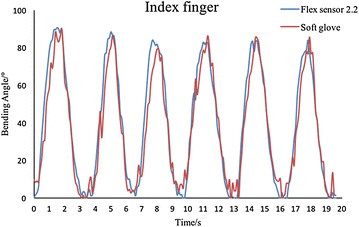
Testing results of MP joint of the soft rubber data-collecting glove. The results have proved the excellent repeatability and precision of the soft sensor glove. And more fluctuation on the *red line* shows that the soft rubber data-collecting glove has better sensitivity than the Flex Sensor 2.2

The bending angle of the sensor can be obtained by solving (13).

Three different sizes of sensors are designed to access the influence of sensor size on repeatability, and the sizes of sensors are $10\times 20\,\hbox {mm}, 10\times 30\,\hbox {mm}$ and $5\times 20\,\hbox {mm}$ respectively. Figure 8 shows the testing results. The average error between the standard value and the measured value are 1.87, 10.53 and 1.46% for the size of $10\times 20\,\hbox {mm}, 10\times 30\,\hbox {mm}$ and $5\times 20\,\hbox {mm}$ respectively. The sensors with the size of $10\times 20\,\hbox {mm}$ and $5\times 20\,\hbox {mm}$ exhibit good repeatability, while the sensor with the size of $10\times 30\,\hbox {mm}$ has a larger error compared with the other two sensors, proving that when the length of the sensor exceeds 20 mm, the repeatability will drop.

### Drifting

According to the sensitivity test and repeatability test, the sensing material with the size of $5\,\hbox {mm}\times 20\,\hbox {mm}$ exhibited the best performance. In this case, we used the size $5\,\hbox {mm}\times 20\,\hbox {mm}$ to test the drifting. we used the same platform in the last subsection to test the drifting. The sensor was flat at the very beginning. After 30 s, the sensor was bent to 60$^{\circ }$ and waited for 30 s. Then, the sensor was bent to 120$^{\circ }$ and waited for 30 s. Then, the sensor was flat again and the whole process was repeated for 5 times. The testing result is shown in Fig. 9.

The testing results show that the sensing material’s resistance will drop when the material is kept in a certain angle. The decrease in resistance is 8.5, 3.1 and 1.5% when then bending angle is 0$^{\circ }, 60^{\circ }$ and 120$^{\circ }$ respectively.

## Glove evaluation

### Fabric data-collecting glove

In order to evaluate the repeatability and precision of fabric data-collecting glove, a commercially available bending sensor, the Flex Sensor 2.2 (Spectra Symbol, Salt Lake City, UT 84119, USA), is used as a contrast to test the fabric sensor glove. Figure 10 exhibits the evaluation of Flex Sensor 2.2, the average error between theoretical value and measured value is 7.1%. In total five flex sensors are used, and they are fixed on PIP joints. The flex sensors and the bending sensors mounted on the glove are firmly attached to each other, so the bending angles of these two sensors can be considered to be the same. Data from flex sensor and bending sensor on the glove are collected at the same time. Figure 11 and Table 2 show the testing results.

The two lines of the index finger match quite well, and each cycle perform almost the same. Same tests were repeated on the other four fingers, and the performance is as good as the index finger. Variation in all the five fingers is less than 7%, illustrating the fabric data-collecting glove has excellent repeatability and precision.

### Soft rubber data-collecting glove

The soft rubber data-collecting glove is made of five parts, and every part has the same structure with a small difference in the length of three belts. In this case, we only exhibit one part of the glove. We choose the part on the index finger to test, and Flex Sensor 2.2 is also used as a contrast. The results are shown in Fig. 12.

The results have shown that the two lines match very well. The visible fluctuations are potentially caused by unstable connections between conduct wire and the sensor elongation. The average error between flex sensor and bending sensor on the soft glove of MP is 7.1%, illustrating high accuracy of the soft rubber data-collecting glove. Compared with traditional fabric gloves, the bionic design of the soft glove makes it possible for directly sensing the bending motion without any medium.

## Conclusions

In the paper, we have presented two different data gloves based on a same soft bending sensor. The sensor is a combination of electrical components and mechanical design. The sensor has excellent sensitivity as well as repeatability. Compared with existing bending sensors, the soft bending sensor is also flexible and can be stretched. The unrecoverable elongation caused by stretch is avoided by a novel structure. The size of the sensor can also be customized. The fabric data-collecting glove solves the problem of sensor slipping, and the size of the glove can be fully customized. The soft rubber data-collecting glove, in which have totally get rid of traditional design methods, provides a new way for making data gloves. The soft glove acts like a layer of personal customized skin on the back of the hand, and bending motion can be measured directly. The modular design of the glove also simplifies the design and manufacturing procedures. Besides, both the sensor and the two kinds of glove have low cost. The production cost of one such bending sensor is less than 2 dollars, and the costs of two kinds of gloves are less than 30 dollars. Such sensing technology plays a huge role in promoting data-collecting devices from science laboratory into clinical use.

In the future, the bending sensor’s repeatability will be improved by reducing hysteresis using an appropriate algorithm. The sensor’s sensitivity with respect to time will be measured. And more bending sensors will be added on a glove. For example, the rotation of thumb will be measured to estimate fingertip distance. We also plan to use this kind of bending sensors to measure the bending angles of wrist and elbow to capture the motion of the whole upper extremity.
